# Frontotemporal Dementia and Amyotrophic Lateral Sclerosis: A Case Report and Clinical Insights

**DOI:** 10.7759/cureus.80329

**Published:** 2025-03-10

**Authors:** Audrey Grigore, Lynne J Goebel

**Affiliations:** 1 Internal Medicine, Joan C. Edwards School of Medicine at Marshall University, Huntington, USA; 2 Internal Medicine and Geriatrics, Joan C. Edwards School of Medicine at Marshall University, Huntington, USA

**Keywords:** als, dementia, ftd, motor neuron disease, tdp-43

## Abstract

Primary care providers are often the first contact for patients with neurodegenerative illnesses, however, they may not be aware of the relationship of certain diseases that may have an impact on their patients' longevity. This case report reminds clinicians of the association between frontotemporal dementia (FTD) and amyotrophic lateral sclerosis (ALS). Physicians should be aware of the association, because FTD commonly occurs first and may prepare clinicians to be alert to the signs of ALS in these patients, leading to earlier detection of ALS and the prescription of disease-modifying medication that may extend the lifespan of people with these diseases. We describe the case of a 61-year-old female patient initially presenting with cognitive decline most likely due to FTD who subsequently developed ALS.

## Introduction

Frontotemporal dementia (FTD) and amyotrophic lateral sclerosis (ALS) are neurodegenerative disorders that affect cognitive and motor cortices respectively [1,2]. FTD is a disease characterized by the degeneration of neurons in the frontal and temporal lobes causing memory problems, personality changes, irritability, executive dysfunction, and decreased ability to carry out activities of daily living [1]. ALS is a motor neuron disorder affecting the loss of upper and lower motor neurons leading to progressive muscle weakness [2]. Although ALS mainly affects the motor system, more severe cases have symptoms of cognitive, behavioral, and language dysfunction meeting the clinical criteria for frontotemporal dementia as well [3]. In fact, 15-20% of patients with ALS also have the behavioral variant of FTD [4]. The causes of the overlap in ALS and FTD are less well-documented, and no curative treatment currently exists for either disease [3]. When they occur together, bulbar manifestations such as facial, tongue weakness, or dysphagia occur more often than in ALS alone [5]. Also, these patients will have pseudobulbar symptoms such as inappropriate laughter or crying [6].

The cause of the association between ALS and FTD includes genetic, clinical, and pathological commonalities [5]. When ALS occurs alongside FTD, the disease progresses more quickly, and survival is significantly reduced compared to ALS alone. ALS and FTD often manifest in individuals during their 50s and are more prevalent in men than in women [1,2]. Studies show that the median survival for individuals with both ALS and FTD is about three years and four months from the onset of symptoms [7]. This markedly reduced survival time highlights the rapid disease progression when ALS and FTD are present together. In comparison, those with ALS alone have a median survival of three to five years and those with FTD alone survive on average 7.5 years [1,2]. By highlighting this case, we aim to underscore the clinical association between ALS and FTD, promoting awareness that may facilitate earlier diagnosis and timely intervention with disease-modifying treatments that could extend survival. Additionally, this case contributes to the growing body of literature on ALS-FTD overlap, emphasizing the need for further research into its common pathophysiology and therapeutic options.

## Case presentation

The patient was a 61-year-old Caucasian female with a history of hypertension and asthma who reported memory trouble at age 48, after her knee surgery. The patient forgot the words to the Doxology while singing in church, a song she had repeated every week for many years. Her son and daughter said she was “different” ever since her knee surgery. She gave irrational answers to questions, was impatient, repetitive, and had rambling speech. It took her longer to read and she had decreased short-term memory. According to family members, her cognitive decline was insidious, with subtle memory lapses and occasional difficulty recalling familiar names and events over the subsequent years. No one in her family had dementia. Her brain computed tomography scan (CT) and thyroid-stimulating hormone levels were normal. Her primary care physician diagnosed her with Alzheimer’s dementia. A depression screen was not found in the records so it is not clear if depression was playing a role in her memory problem at this point. The physician initially prescribed donepezil, which she did not tolerate, and later changed her to the rivastigmine patch, which she tolerated well. The patient thought her memory improved on this medication. She was able to perform all activities of daily living (ADLs), and instrumental activities of daily living (IADLs) independently as documented in a follow-up note while on the medication. Over the next several years, she was noted to be irritable at work, talking quickly and having trouble with boundaries, suffering from anxiety, and having waxing and waning memory testing.

The patient presented to a geriatric specialist at the Hanshaw Geriatric Center affiliated with Marshall University five years after her initial diagnosis. At that time, she scored 10/10 on her clock drawing test and 30/30 on a Folstein Mini-mental Status Examination (MMSE). She used lists more than ever and wrote down recipes that she always knew by heart. She was very active in a women’s club. She noted that she needed quiet to concentrate on reading. She still used the rivastigmine patch. She did not have any behavioral manifestations or speech difficulties. The new doctor tapered her off the patch and a year later her memory remained stable.

Eight months later, however, the patient had a cholecystectomy and noticed a decline in her memory since the surgery. She had problems with word finding and repetitiveness. She became frustrated, easily lost her train of thought, and did not read as much as she did before the surgery. Her personality changed, and she became bossy. She had difficulty with speech and showed a marked decline since her last visit. At the visit shortly after the operation, she scored 23/30 on the MMSE, indicating abnormal cognitive function (normal: 26-30), with pronounced deficits in attention and recall. The MMSE assesses global cognitive function, including orientation, memory, attention, language, and visuospatial abilities. She also scored 8/10 on the clock draw test, which evaluates executive function and visuospatial processing. A brain CT scan was unremarkable, ruling out acute structural abnormalities as a cause of her cognitive decline. The patient resumed the rivastigmine patch. After a month, her confusion resolved. She scored 27/30 on a Montreal Cognitive Assessment (MoCA) test. The MoCA test is used more often in memory evaluation clinics to test cognitive function and takes longer to administer than the MMSE. A normal score is over 26. Unlike the MMSE, the MoCA emphasizes executive function, attention, and delayed recall, making it particularly useful for detecting early cognitive impairment. Her improved MoCA score suggested a transient component to her cognitive symptoms, potentially related to post-surgical stress or medication effects.

Over the next five years, her memory was noted to be stable except when she missed changing her patch. She remained independent of her ADLs and IADLs and reported no emotional changes. She had a new geriatric provider who thought she had mild cognitive impairment but continued the rivastigmine as the patient still thought she got worse when not using the patch.

A week after establishing with this new provider and 12 years after her initial memory complaints, the patient started tripping due to right foot weakness. She dragged her toes and could not lift the forefoot off the ground. The patient also complained of non-radicular lower back pain which worsened with trunk extension and improved with flexion. In addition, she noticed some proximal weakness in the right lower extremity and right upper extremity. She now required assistance in doing most ADLs which were not problematic in the past. She used a walker for ambulation. The geriatrician consulted neurology. On physical exam, she had 4/5 weakness in the right upper extremity, 3/5 proximal right leg weakness, and 0-1/5 weakness in the right foot. No fasciculations were noted. Muscle tone and bulk were normal except for some subtle atrophy of the right first dorsal interosseous muscle. Reflexes were slightly less in the right Achilles tendon and hyperreflexic elsewhere. Sensation remained intact.

Her medications were albuterol inhaler, naproxen, fexofenadine, amlodipine, aspirin, calcium and vitamin D tablets, candesartan/hydrochlorothiazide, multivitamin, azelastine hydrochloride, rivastigmine patch, and montelukast.

Please see the timeline for a summary of her presentation in Table 1.

**Table 1:** Timeline of case presentation

Year	Event
Age 48	Memory trouble reported after knee surgery; forgot familiar song; personality changes noted.
Follow-up Years	Irritability, rambling speech, and decreased short-term memory. Initial diagnosis: Alzheimer’s Dementia.
5 years later	Evaluated at a Geriatric specialist. Normal MMSE (30/30) and clock drawing (10/10). Rivastigmine patch improved symptoms.
One year later	Memory remained stable despite tapering off rivastigmine.
8 months later	Underwent cholecystectomy. Cognitive decline with word-finding difficulties, speech changes, and increased assertiveness.
1 month post-surgery	Restarted rivastigmine. MoCA improved to 27/30. ADLs and IADLs stable.
Following 5 years	Memory remained stable with rivastigmine, except when missed doses.
Age 61	Developed right foot weakness, toe dragging, proximal limb weakness. Required walker. Consulted Neurology.
Neurological Exam	EMG/NCS showed denervation with fasciculations. Diagnosed with clinically definite ALS.
Further Evaluation	Brain MRI unremarkable; lumbar MRI showed mild degenerative changes. Diagnosis of ALS-FTD suspected.
Final Diagnosis	ALS with possible FTD based on cognitive and motor decline, personality changes, and fluctuating executive function.
Treatment Considerations	Riluzole and edaravone suggested for ALS; SSRIs considered for behavioral symptoms of FTD.

**Figure 1:**
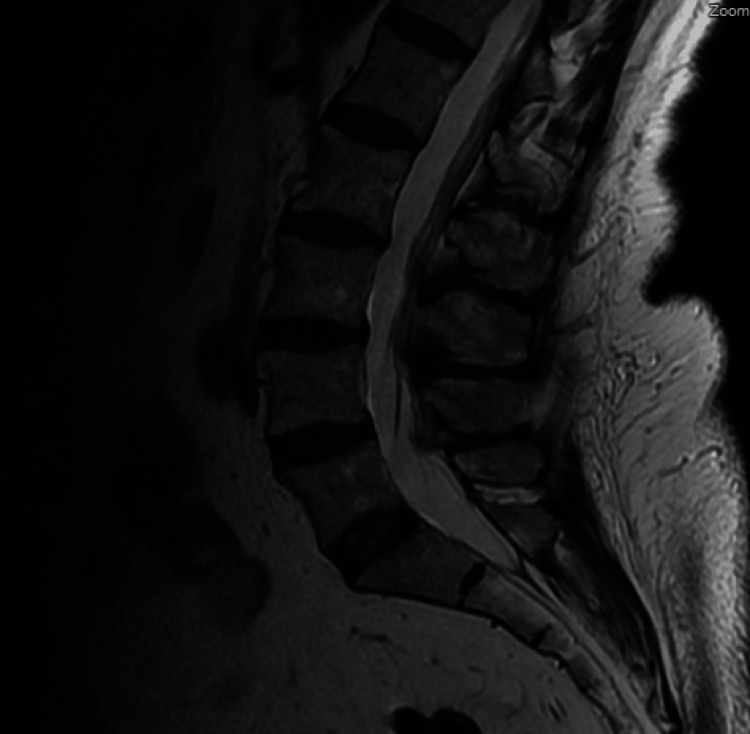
MRI lumbar spine showing minimal degenerative changes

A magnetic resonance imaging study (MRI) of the lumbar spine (Figure 1) showed minimal multilevel degenerative changes with facet arthropathy and minimal disc bulging. These suggest arthritic and degenerative findings and are not the likely cause of her weakness.

Table 2 reviews the findings of our case and how they relate to ALS and FTD. 

**Table 2:** Clinical and radiologic features of ALS and FTD in our patient ALS: amyotrophic lateral sclerosis; FTD: frontotemporal dementia

Symptoms and testing modalities	Findings in the patient	Relevance to ALS-FTD
Cognitive symptoms	Memory impairment, word-finding difficulty, personality changes	FTD often presents with early behavioral and executive dysfunction
Motor symptoms	Progressive weakness, speech difficulty	ALS primarily affects upper and lower motor neurons, leading to muscle weakness and dysarthria
Behavioral changes	Increased frustration, bossiness	Behavioral variant FTD (bvFTD) is common in ALS-FTD, characterized by disinhibition and personality changes
Radiological findings	Minimal lumbar degenerative changes	Not directly related to ALS-FTD, but rules out spinal pathology as the cause of symptoms
Neuroimaging (CT/MRI Brain)	Brain CT: Unremarkable	A brain MRI may be more informative, as FTD typically shows frontal and temporal lobe atrophy

Electromyography and nerve conduction studies (EMG/NCS) revealed both acute and chronic denervation/reinnervation evidence in multiple muscles of different limbs, mid-thoracic and lumbar paraspinal regions. Fasciculations involving the right upper and lower extremities, and tongue muscles were prominent. These findings point to denervation affecting the face, upper limbs, and lower limbs. 

Vitamin B12, creatinine phosphokinase, thyroid stimulating hormone, hemoglobin A1c, and erythrocyte sedimentation rate were normal. Anti-myelin-associated glycoprotein and antiganglioside antibodies were also negative. She was diagnosed with clinically definite ALS. 

The patient received riluzole disease-modifying treatment. Over the following year, she continued to experience progressive respiratory impairment, requiring non-invasive ventilatory support. She refused tracheostomy and further invasive treatment, received hospice care and died of respiratory failure two years after her symptoms of ALS began.

## Discussion

Our patient most likely had both FTD and ALS. Numerous case reports document the association between these two conditions. Various diagnostic methods are available for each disease, and they share certain protein and genetic overlaps. We will discuss the association between the two diseases, the diagnosis, and treatment options.

One potential reason for the overlap of these two diseases is due to aggregates of transactive response DNA-binding protein 43 (TDP-43 proteins) or tau proteins that are misfolded in glial and neuronal inclusion bodies occurring in the brain [8]. TDP-43 is a pathological protein associated with frontotemporal lobar degeneration (FTLD). FTLD is often used interchangeably with FTD, however, FTLD refers to a larger group of disorders with FTD being one of its subgroups [9]. In nearly half of all FTLD cases, TDP-43 is a key contributor with abnormal tau protein [8]. FTLD-TDP is characterized by significant TDP-43 pathology in the frontotemporal cortices. This pathology also affects the limbic system, hippocampus, neostriatum, and substantia nigra [10].

Not only does TDP-43 accumulate in FTLD, but it does similar things in the neurons of ALS patients. TDP-43 mislocalizes from the nucleus and forms cytoplasmic aggregates in affected motor neurons in ALS [10]. Over 90% of sporadic ALS cases exhibit TDP-43 pathology. Autopsy-based studies show that TDP-43 pathology in ALS follows a corticofugal descending pattern, starting from the primary motor cortex and extending to postsynaptic neurons, including lower motor neurons, striatal neurons, and other regions in the frontal and temporal lobes [8]. This pattern of progression aligns with our patient’s disease course, as she initially presented with subtle cognitive deficits and later developed progressive motor weakness, including right foot drop, proximal limb weakness, and hyperreflexia, which ultimately led to her ALS diagnosis.

The mechanisms leading to protein aggregates in sporadic cases of TDP-43 proteinopathies or tauopathies are not well understood, and most neurodegenerative disorder cases are sporadic [11]. The pathogenesis of these disorders cannot be attributed to alterations in a single gene or molecule [8]. Our patient’s family history does not indicate inherited ALS or FTD. Therefore, it is likely that this is a sporadic case. Geriatricians are more familiar with aging-related TDP-43 pathology, prominent in the limbic system, known as limbic-predominant age-related TDP-43 encephalopathy (LATE) [12]. Studies have demonstrated that neuropathologists can distinguish between LATE and the TDP-43 pathology in ALS/FTLD-TDP with high sensitivity and specificity, due to the broader distribution and higher density of cortical TDP-43 pathology in ALS and FTLD-TDP [8]. Our patient was relatively young, and her differential diagnosis included early Alzheimer's disease as well as FTD, but LATE was not considered due to it occurring more often in people over age 80.

In looking at possible genetic associations between FTD and ALS, inherited and environmentally caused mutations in the genes C9orf72, TARDBP, FUS, TBK1, VCP, CHCHD10, and SQSTM1 are closely linked with both diseases [13]. Mutations of the genes C9orf72, GRN, MAPT, and TMEM106B encode proteins that have downstream effects on endosome-lysosome function and can lead to the pathology of both FTD and ALS [14]. Most of these genes are involved in RNA metabolism, protein homeostasis, autophagy, or mitochondrial function--all crucial for maintaining neuronal health. Their mutations lead to protein aggregation, impaired degradation, or mitochondrial dysfunction, contributing to neurodegeneration in ALS and FTD. Although our patient did not undergo genetic testing, her clinical course fits the pattern observed in sporadic cases of ALS/FTD with TDP-43 pathology.

The diagnosis of ALS is primarily based on clinical evaluation using the El Escorial criteria, which classifies the disease from possible ALS to definite ALS [15]. Four anatomical regions are assessed: bulbar, cervical, thoracic, and lumbar. The presence of upper and lower motor neuron signs determines the severity of ALS. EMG and NCS, MRI and CT scans of the brain and spine, identification of biochemical markers in blood and cerebrospinal fluid, muscle or nerve biopsy, and genetic testing confirm the diagnosis and rule out other conditions. Genetic testing is useful for detecting gene mutations in familial ALS and other inherited motor neuron diseases that are similar to ALS. Our patient was diagnosed with definite ALS when she had the classic EMG/NCS findings and a negative MRI of the brain and spine.

The diagnosis of FTD is primarily made clinically. Patients are generally not a reliable source of behavioral problems therefore it is important to interview family members. The presence of apathy, eating disorders, atypical social conduct, and compulsive behaviors associated with the absence of visual spatial or memory deficits are specific and sensitive for the diagnosis of FTD [16]. Possible FTD requires three of the six clinical features: disinhibition, apathy/inertia, loss of sympathy/empathy, perseverative/compulsive behaviors, hyperorality, or dysexecutive neuropsychological profile [16]. Probable FTD requires the same clinical criteria with specific neurodegeneration in neuroimaging findings, such as frontal or temporal lobe atrophy, hypometabolism, hypoperfusion, and functional decline [17]. Our patient likely has possible FTD due to meeting at least three of the six criteria for FTD. To support the diagnosis of FTD, MRI and CT scans were used to exclude other potential differential diagnoses including vascular dementia. Depression and psychiatric illness could cause some of her symptoms and neuropsychiatric evaluation could be considered if there was a question about her diagnosis. FTD can be confirmed with possible or probable FTD when analyzed by histopathology at autopsy or the presence of a known pathogenic mutation [17]. Our patient had a change in her personality. This may be consistent with FTD. Her clinical course was not classic in that she had memory problems that waxed and waned and seemed related to her surgery at first making delirium a possible diagnosis. She also had waxing and waning problems with her clock drawing, which is the measure of executive function that is usually preserved in FTD but lost earlier in Alzheimer's disease which was the main alternative diagnosis considered.

Patients with FTD often worsen with cholinesterase inhibitors [18]. Our patient initially worsened with donepezil, however, she improved after switching to rivastigmine, a medication in the same class. Her brain CT scan did not show the atrophy in the frontal and temporal lobes seen in FTD but this does not rule out the disease. FTD usually occurs in people younger than 65 years of age and progresses with time leading to cognitive and behavioral changes that affect ADLs [1]. Our patient was the typical age for FTD when she first had memory problems. However, our diagnosis is limited due to incomplete documentation in the medical record. After her cholecystectomy, the patient had symptoms that could be due to FTD such as problems with word finding, repetitiveness, frustration, reasoning, speaking, and reading. Later, she could not perform her ADLs independently indicating progression to dementia. 

There are potential treatments and management options for FTD and ALS. Early diagnosis of ALS in patients with FTD is crucial for increasing patients' longevity and quality of life. Currently, no approved disease-modifying treatments for FTD exist, but medications are available to manage symptoms. In FTD patients, selective serotonin reuptake inhibitors (SSRIs) such as sertraline, paroxetine, and fluoxetine can help reduce disinhibition, impulsivity, and compulsive behaviors. FDA-approved disease-modifying drugs like riluzole and edaravone are available to slow the progression of ALS. In a clinical trial, riluzole, at a dose of 100 mg, increased the survival rate for ALS by two to three months with a 35% decreased risk of death [19]. Another clinical trial showed that intravenous edaravone prolonged the median survival of ALS patients by six months and decreased mortality by 27% [20]. While these drugs do not cure ALS, they slow the disease, which improves longevity and possibly adds to quality of life. Future research exploring gene therapy as a potential treatment for ALS is on the horizon.

## Conclusions

This case highlights the association between FTD and ALS, linked by misfolded tau proteins and genetic factors. Patients presenting with personality changes and memory complaints should be monitored for motor neuron disease, as early intervention with disease-modifying agents may slow ALS progression. Observed data in this case demonstrated cognitive decline, episodic worsening post-surgery, and eventual motor symptoms, emphasizing the need for a multidisciplinary approach. Healthcare providers should incorporate cognitive and motor assessments in follow-up care for suspected FTD cases, particularly when symptoms fluctuate or progress rapidly. Future research should focus on predictive markers and targeted treatments, including gene therapy, to address the underlying pathology of both diseases.
